# Unusual Appearance of a Pendulated Gastric Tumor: Always Think of GIST

**DOI:** 10.1155/2012/815941

**Published:** 2012-09-04

**Authors:** Kristel De Vogelaere, Vanessa Meert, Frederik Vandenbroucke, Georges Delvaux, Anne Hoorens

**Affiliations:** ^1^Department of Abdominal Surgery, UZ Brussel, Laarbeeklaan 101, 1090 Brussels, Belgium; ^2^Department of Pathology, OLV Aalst, Moorselbaan 163, 9300 Aalst, Belgium; ^3^Department of Radiology, UZ Brussel, Laarbeeklaan 101, 1090 Brussels, Belgium; ^4^Department of Pathology, UZ Brussel, Laarbeeklaan 101, 1090 Brussels, Belgium

## Abstract

*Objective*. To investigate the clinicopathological characteristics of gastrointestinal stromal tumor (GIST) with significant cystic changes and to assess the molecular genetic characteristics. *Methods*. In a 68-year-old man, a large abdominal tumoral mass was discovered incidentally. Computed tomography (CT) and magnetic resonance imaging (MRI) confirmed the presence of a large cystic lesion with multiple contrast-enhancing septae and papillary projections. No clear connection with any of the surrounding organs was identified. Malignancy could not be excluded, and surgery was indicated. During surgery, the large mass was found to be attached by a narrow stalk to the large curvature of the stomach. *Results*. The histological features and immunohistiochemical profile of the tumor cells (positivity for CD117 and CD34) were consistent with a gastrointestinal stromal tumor with a high risk of progressive disease according to the Fletcher classification. Diagnosis was confirmed by mutational analysis; this demonstrated mutation in exon 14 of PDGFRA. During the followup of 97 months, the patient had a cancer-free survival. *Conclusions*. This case demonstrates that gastrointestinal stromal tumors (GISTs) with extensive cystic degeneration should be considered in the differential diagnosis of a cystic abdominal mass.

## 1. Introduction

Gastrointestinal stromal tumors (GISTs) are specific mesenchymal tumors of the gastrointestinal tract. GISTs are rare, accounting for only 0.2% of all gastrointestinal tumors [1]. Morphologically they show similarities to other tumor types and were previously misclassified as leiomyomas, leiomyoblastomas, or leiomyosarcomas, but also as schwannomas or malignant peripheral nerve sheath tumors. Since the discovery of KIT (CD117) in 1998, GISTs were identified as a distinct entity [2]. These tumors are believed to originate from the interstitial cells of Cajal or related stem cells [3–6]. Proper identification of GIST has become very important since the availability of a specific pathogenesis-targeted treatment, namely, imatinib. GISTs usually present as solid tumors. We report an incidental finding of a cystic gastric GIST with exophytic pedunculated growth.

## 2. Case Report

In a 68-year-old man, a large abdominal tumoral mass was discovered incidentally during checkup for vascular insufficiency of the lower limbs. The patient complained of vascular insufficiency and had no symptoms of abdominal pain. Physical examination revealed a palpable mass in the right upper quadrant of the abdomen. Results of blood samples were all within normal values.

**Figure 1:**
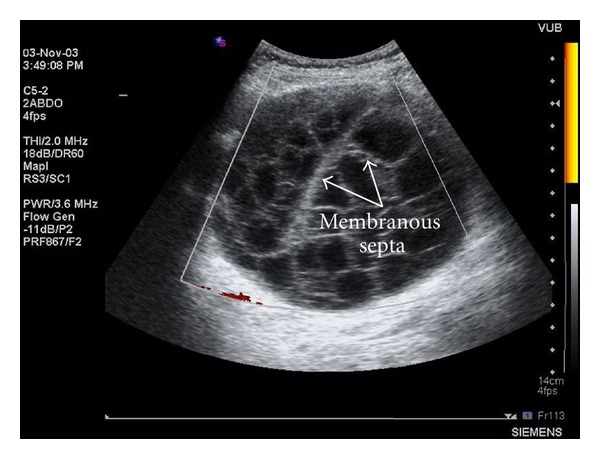
Abdominal ultrasonography (US) showing a well-defined large cystic mass in the right hypochondrium with several membranous septa (arrow).

**Figure 2:**
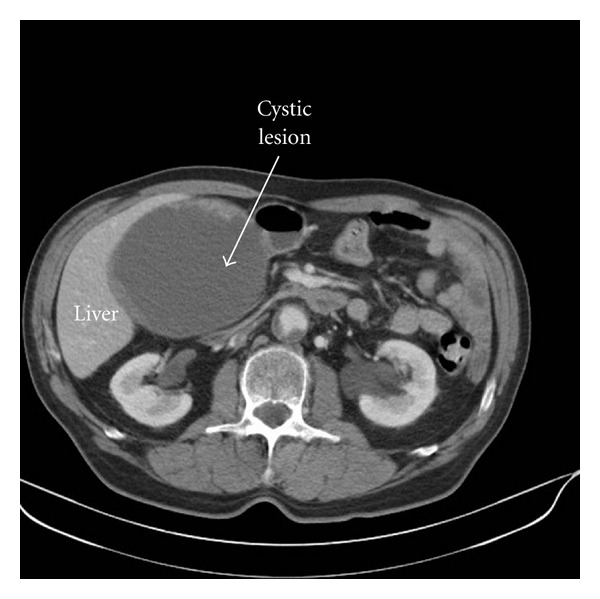
Abdominal computed tomography (CT) displaying a large cystic lesion (arrow) adjacent to the right liver lobe, the stomach, and the pancreatic head.

**Figure 3:**
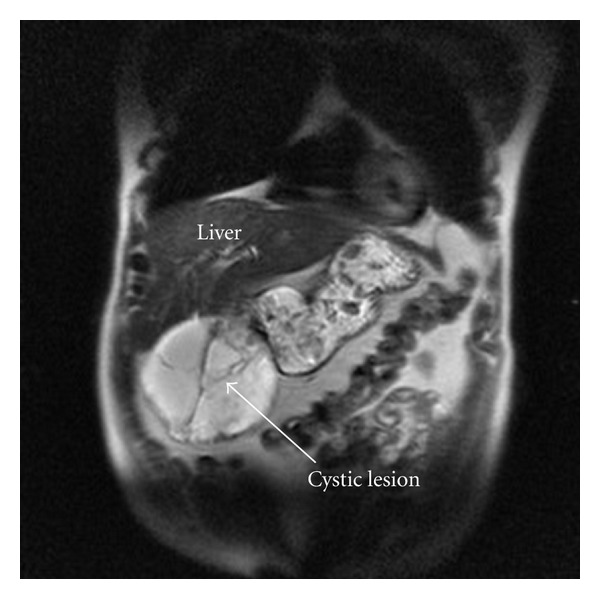
On MRI imaging markedly high intensity, compatible with cystic nature (arrow), was revealed in the tumor.

Ultrasonography showed a well-defined large cystic mass with several membranous septa with a diameter of approximately 12 cm in the right hypochondrium (Figure 1). Subsequent computed tomography (CT) scan demonstrated a large cystic lesion adjacent to the right liver lobe, the stomach, and the pancreatic head (Figure 2). On MRI imaging, markedly high intensity, compatible with cystic nature, was revealed in the tumor. The tumor was lying in contact with the right liver lobe, the stomach, and the pancreatic head (Figure 3).

However, no clear connection with any of these organs was identified. The imaging findings demonstrated no vascularity of the lesion. Malignancy could not be excluded, and surgery was indicated.

At laparotomy a large exophytic mobile mass was found to be attached by a narrow stalk to the larger curvature of the stomach at the level of the antrum. No infiltration into the surrounding tissues was observed.

Surgical resection of the mass along with a wedge resection of the stomach, at the larger curvature, adjoining the stalk and the adherent omentum was performed.

**Figure 4:**
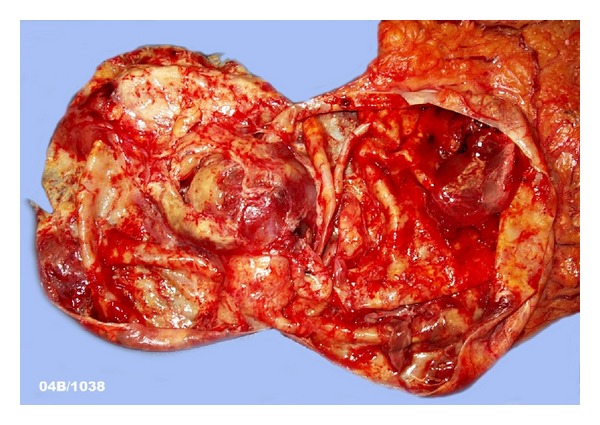
Macroscopically, a large unilocular cystic tumor with a smooth outer surface and several membranous septa projecting in the lumen was found.

The resected tumor measured almost 12 cm in diameter. Grossly, it was a unilocular cystic tumor filled with serous fluid, with a smooth outer surface and several membranous septa projecting in the lumen (Figure 4). The cyst had a thin wall. The stalk was connecting the cystic tumor with the stomach. The mitotic activity was less than 5 mitoses per 50 HPF. The tumor cells showed positivity for CD117 (KIT) and CD34. The histological features together with the immunohistiochemical profile of the tumor cells were consistent with a gastrointestinal stromal tumor (GIST) with a high risk of progressive disease according to the Fletcher classification. Molecular analysis of this tumor showed a mutation in exon 14 of the PDGFRA gene (substitution exon 14, N659 K). Since PDGFRA exon 14 mutant GIST is sensitive to imatinib mesylate (Glivec), this oral treatment was started postoperatively because the high risk of recurrence in this patient (high risk of progressive disease according to the Fletcher classification). Untill now the patient is still free of recurrence.

## 3. Discussion

Gastrointestinal stromal tumors (GISTs) are rare neoplasms, with an annual incidence of approximately 4 per million [3]. Historically, these tumors were classified as leiomyomas, leiomyoblastomas, and leiomyosarcomas, because of a mistaken belief that they originated from smooth muscle in the wall of the gastrointestinal tract [2].

The cellular origin of GIST recently has been proposed to be the interstitial cell of Cajal, an intestinal pacemaker cell. This postulate is supported by the finding that GISTs display positivity for cell markers similar to those of the normal cell of Cajal [4–6]. The majority (approximately 95%) of GISTs express the CD117 antigen (KIT), a protooncogene product. CD34, a commonly expressed human progenitor cell antigen, is also frequently found positive in GISTs [3]. More than half of the GISTs are located in the stomach followed, by the small intestine, colon and rectum, and esophagus [1, 3, 7].

Complete tumor resection with disease-free resection margins is the treatment of choice for primary nonmetastatic tumors. Lymphadenectomy is not recommended because lymph node involvement is rare. Wedge resection allows full-thickness resection of the stomach wall containing the tumor, with negative resection margins [1, 6].

Grossly, GISTs vary greatly in size and can be more than 30 cm in diameter. These tumors are usually well circumscribed and unencapsulated. GIST can grow in an endophytic or exophytic pattern. They are usually solid. Small cysts are frequently observed, presumably as a consequence of cystic degeneration or necrosis. Larger stromal tumors usually degenerate, and cysts are formed [8–11].

In the present case, the large size of the cyst obscured the origin from the stomach. Imaging showed that the tumor was not originating from the pancreas or any other organ, so the exact origin of the tumor could not be determined preoperatively. Imaging demonstrated no vascularity of the lesion. Since malignancy could not be excluded in our case and the origin of the tumor could not be determined by imaging, surgery was indicated.

Complete tumor resection with disease-free resection margins is the treatment of choice for primary nonmetastatic tumors. Lymphadenectomy is not recommended because lymph node involvement is rare. Wedge resection allows full-thickness resection of the stomach wall containing the tumor, with negative resection margins [1, 6].

Lesions that should be considered in the differential diagnosis of a cystic abdominal mass on radiologic imaging (CT and MRI) include gastric or bowel duplication cysts, cystic mesothelioma, cystic lymphangioma, cystic mucinous retroperitoneal tumors, cystic pancreatic tumors, pseudocysts of the pancreas or peritoneum, cystic teratoma, and GIST [12–14].

In this case, tumor cells showed diffuse and strong positivity for CD117 (KIT) and CD34, which was consistent with a diagnosis of GIST. This was confirmed by molecular biology that showed a mutation in exon 14 of the PDGFRA gene (exon 14 substitution, N659 K).

Wang et al. recently published a series of 7 patients with cystic GISTs and analysis of c-kit and PDGFRA gene. Gene mutation of exon 11 of c-kit was identified in 3 cases [15]. PDGFRA mutant GISTs arise almost exclusively in the stomach, whereas KIT mutant tumors occur at a variety of sites along the gastrointestinal tract. PDGFRA exon 14 mutations may be associated with a reduced risk of recurrence. Limited clinical data are published, but PDGFRA exon 14 mutant GISTs appear; sensitive to imatinib, the sensitivity is similar to KIT exon 11 mutants [16–18].

In summary, GISTs with cystic appearance clearly should be considered in the differential diagnosis of cystic abdominal tumors. Most GISTs (95%) express Kit (CD117) and CD34 (70%). In case of doubt genmutation analysis is necessary. KIT and PDGFRA genotyping is important for GIST diagnosis and assessment of sensitivity to tyrosine kinase inhibitors.
